# Cordycepin delays postovulatory aging of oocytes through inhibition of maternal mRNAs degradation via DCP1A polyadenylation suppression

**DOI:** 10.1007/s00018-023-05030-0

**Published:** 2023-11-25

**Authors:** Chong Li, Ling Zhu, Jun-Xia Liu, Jing Guo, Juan Xie, Chun-Meng Shi, Qing-Yuan Sun, Guo-Ning Huang, Jing-Yu Li

**Affiliations:** 1 Chongqing Key Laboratory of Human Embryo Engineering, Center for Reproductive Medicine, Women and Children’s Hospital of Chongqing Medical University, Chongqing, China; 2 Chongqing Clinical Research Center for Reproductive Medicine, Chongqing Health Center for Women and Children, Chongqing, China; 3 College of Animal Science and Technology, Jilin Agricultural University, Changchun, China; 4 Institute of Rocket Force Medicine, State Key Laboratory of Trauma, Burns and Combined Injury, Third Military Medical University, Chongqing, China; 5 Guangzhou Key Laboratory of Metabolic Diseases and Reproductive Health, Reproductive Medicine Center, Guangdong Second Provincial General Hospital, Guangzhou, China

**Keywords:** Postovulatory aging, Maternal mRNAs, Cordycepin, DCP1A

## Abstract

Postovulatory aging leads to the decline in oocyte quality and subsequent impairment of embryonic development, thereby reducing the success rate of assisted reproductive technology (ART). Potential preventative strategies preventing oocytes from aging and the associated underlying mechanisms warrant investigation. In this study, we identified that cordycepin, a natural nucleoside analogue, promoted the quality of oocytes aging in vitro, as indicated by reduced oocyte fragmentation, improved spindle/chromosomes morphology and mitochondrial function, as well as increased embryonic developmental competence. Proteomic and RNA sequencing analyses revealed that cordycepin inhibited the degradation of several crucial maternal proteins and mRNAs caused by aging. Strikingly, cordycepin was found to suppress the elevation of DCP1A protein by inhibiting polyadenylation during postovulatory aging, consequently impeding the decapping of maternal mRNAs. In humans, the increased degradation of DCP1A and total mRNA during postovulatory aging was also inhibited by cordycepin. Collectively, our findings demonstrate that cordycepin prevents postovulatory aging of mammalian oocytes by inhibition of maternal mRNAs degradation via suppressing polyadenylation of DCP1A mRNA, thereby promoting oocyte developmental competence.

**Supplementary Information:**

The online version contains supplementary material available at 10.1007/s00018-023-05030-0.

## Introduction

Following ovulation, oocytes arrest at the metaphase of the second meiosis (MII), awaiting fertilization. If fertilization does not occur in time, oocyte quality incrementally deteriorates over time, known as postovulatory aging [1, 2]. Over the past three decades, assisted reproductive technology (ART) has been extensively employed as an effective intervention for infertility [3]. However, the decline in oocyte quality due to postovulatory aging, especially when rescue intracytoplasmic sperm injection (ICSI) was performed due to fertilization failure during in vitro fertilization (IVF) [4, 5], impairs fertilization and developmental potential, which is one of the prime causes of failure in ART procedure [6–10].

Numerous studies have demonstrated that oxidative stress, altered Ca^2+^ homeostasis, imbalanced redox regulation and mitochondrial dysfunction, induced by postovulatory aging, are major causes for the impaired developmental competence [7, 11–14]. However, the exact molecular mechanism underlying these aging-induced defects still remain largely unknown. Maternal effect genes (MEGs) play crucial roles in various processes such as oocyte maturation, fertilization, oocyte to embryo transition (OET) and zygotic genome activation (ZGA) [15–18]. Therefore, disruption of MEGs would affect the subsequent embryonic development. Recent research shows that postovulatory aging leads to the decrease of poly(A) mRNAs [7, 19]. Moreover, postovulatory aging of mouse oocytes results in degradation of multiple MEG mRNAs, including *Nlrp5* (NLR family, pyrin domain containing 5; also known as MATER), and *Brg1* (SWI/SNF related, matrix associated, actin dependent regulator of chromatin) [19]. The storage of MEG mRNA depends on mRNA-binding proteins like MSY2 (also termed YBX2), which has a crucial role in regulating mRNAs stability [20–23]. Postovulatory aging can lead to the loss of MSY2, which may further accelerate mRNAs degradation [7]. Therefore, preventing this degradation may serve as a strategy to delay the postovulatory aging of oocytes.

Cordycepin, a natural derivative of adenosine also known as 3’-deoxyadenosine, possesses several biological activities, including anti-oxidant [24, 25], anti-inflammation [26], and anti-tumor properties [27]. Our previous study indicated that cordycepin can prevent radiation ulcer by inhibiting cell senescence via NRF2 and AMPK in rodents [28]. Additionally, cordycepin can enhance male reproduction via stimulating steroidogenesis in mouse Leydig cells through the activation of PKA pathway [29, 30]. It has been shown that cordyceptin possesses the ability to inhibit polyadenylation, thereby suppressing RNA synthesis [31]. Considering the fact that oocyte aging and developmental competence are closely related to maternal mRNA changes, we supposed that cordycepin may improve the quality and developmental potential of oocytes aging in vitro.

In this study, we showed that cordycepin inhibited the polyadenylation of DCP1A, which suppresses the degradation of maternal mRNAs, subsequently delaying postovulatory aging. Our findings provide an important approach for improving the quality of oocytes aging in vitro, which may serve as a foundational basis for optimizing assisted reproductive technology (ART), especially when rescue ICSI is employed.

## Materials and methods

### Animals

Institute of Cancer Research (ICR) mice (females: 6–8 weeks old; males: 10–12 weeks old) were employed in this study. The mice were housed in a temperature- and light-controlled room with free access to food and water under a photoperiod of 12 h-light and 12 h-dark. All animals were treated in accordance with the guidelines of the Chongqing Health Center for Women and Children Animal Care and Use Committee (reference number: 2021012).

## Oocyte collection and postovulatory aging

Female mice were injected intraperitoneally with 10 IU of pregnant mare serum gonadotropin (PMSG; Sigma-Aldrich, St. Louis, MO, USA), followed by injection of 10 IU of human chorionic gonadotropin (HCG; Sigma-Aldrich) 48 h later. The superovulated mice were killed 13–15 h after hCG injection and cumulus-oocytes complexes were collected from the oviduct ampulla, placed in 37 ℃ M2 (M7167, Sigma-Aldrich, MO, USA) medium, then cumulus cells were removed by 0.3% hyaluronidase (H4272, Sigma-Aldrich) treatment in M2 medium. Fresh MII oocytes, collected at 13–15 h post-hCG with the first polar body, were selected for the next experiments. They were placed in CZB (M1650, Nanjing Aibei, China) medium under mineral oil (Vitrolife, Göteborg, Sweden), at 37 °C with 5% CO_2_ in the air for in vitro aging. The fresh oocytes without any treatment were used as control in the study.

In human, immature GV oocytes were immediately subjected to in vitro culture in 50 μL of G1-PLUS (Vitrolife) medium, maintained at 37 °C with 5% CO_2_ in the air for 16 h. Then, the MII oocytes with the first polar body were cultured in G1-PLUS medium with DMSO (1:500) or with cordycepin (400 μM) for 24h to mimic postovulatory aging. The patients who donated embryos were between 20 and 35 years old without a history of genetic diseases or smoking. The Institutional Review Board (IRB) of Chongqing Health Center for Women and Children approved this study (reference number: 2022-RGI-13). Donor couples provided signed informed consent at the Center for Reproductive Medicine in The Women and Children’s Hospital of Chongqing Medical University before voluntarily donating the oocytes.

## Drug treatments

Cordycepin (MCE, New Jersey, USA) and U0126 (MCE) was dissolved in DMSO (Sigma-Aldrich) to prepare a stock solution (200mM) and diluted to working concentration with medium before using. The concentrations of cordycepin used in the experiment were 200 μM, 400 μM and 800 μM respectively and the concentrations of U0126 used in the experiment were 1 and 3 μM. The culture medium containing 0.2% DMSO servered as the negative control in this study.

## Intracytoplasmic sperm injection and embryo culture

The injection pipettes had an internal diameter of 8–10 μm at the tip and were siliconized before use. We used a Piezo sperm injection instrument (PiezoXpert, Eppendorf, Germany). Groups of 15 to 20 oocytes were placed in M2 medium with cytochalasin B (5 μg/mL, Sigma-Aldrich). The sperm tails were removed by Piezo pulses, and only one randomly selected sperm head was injected into each oocyte. After 15 min of recovery, the surviving zygotes were washed 3 times in KSOM (Sigma-Aldrich) medium covered with mineral oil and incubated at 37 °C with 5% CO_2_ in the air. Six hours after intracytoplasmic sperm injection (ICSI), pronucleus formation was examined.

## Parthenogenetic activation

Oocytes were activated by SrCl_2_ treatment for 6 h, using a calcium-free CZB medium containing 10 mM SrCl_2_ (Sigma-Aldrich) and 5 mg/mL cytochalasin B. At the end of the 6 h culture, oocytes were observed under an inverted microscope for activation. Oocytes showing one or two pronuclei were considered activated.

## Time-lapse monitoring of embryo morphokinetics

For time-lapse imaging the Vitrolife Embryoscop System (Vitrolife) was used. T0 is the time point when two pronuclei fading (tPNf). Time t2 is interval from syngamy (2PN fading) to the 2 cell stage. Times at t3, t4, t5, t8, tM, tBL are the times of division into 3-, 4-, 5-, and 8-cell embryos, morula, and blastocysts, respectively.

## Detection of protein synthesis

Oocytes were incubated in CZB medium containing 100 mM L-homopropargylglycine (HPG) for 1 h and then fixed for 30 min at room temperature in 4% paraformaldehyde (PFA, Sigma-Aldrich). HPG signals were detected using a Click-iT HPG Alexa Fluor Protein Synthesis Assay Kit (Life Technologies). Mean intensity of the HPG signal was measured across the middle of each oocyte and quantified using ImageJ software.

## Immunofluorescence staining and confocal microscopy

After removing the ZP with 0.5% HCl in phosphate-buffered saline (PBS, pH 7.4), oocytes were fixed with 4% PFA in PBS-PVA for 30 min and then washed 3 times with wash buffer (PBS containing 0.1% Tween 20 and 0.01% Triton X‐100). After that, oocytes were permeabilized for 20 min with 0.5% Triton X-100 in PBS at room temperature. Then, samples were blocked with 3% BSA in PBS for 1 h and incubated with primary antibody (anti-α-tubulin (FITC) antibody, Sigma-Aldrich, F2168, 1:500 dilution; anti-AMPK alpha 1 (phospho T183) + AMPK alpha 2 (phospho T172) antibody, Abcam, ab133448, 1:200 dilution; anti-MOS antibody, Abcam, ab171937, 1:200 dilution; anti-p-MAPK3/1 antibody, Cell Signaling Technology, 4370S, 1:200 dilution; anti-DCP1A antibody, Abcam, ab183709, 1:200 dilution) at 4 °C overnight. After washing 3 times with wash buffer, samples were incubated with second antibody (anti-rabbit IgG (H + L) cross-adsorbed secondary antibody, Alexa Fluor™ 488, Invitrogen, A11008, 1:500 dilution) for 1 h at room temperature. Then samples were counterstained with 10 μg/ml of Hoechst 33,342 (Sigma-Aldrich) for 10 min. Finally, samples were mounted on glass slides and observed under a laser scanning confocal microscope. MitoTracker Red CMXRos (200 nM, Life Technologies CA, USA) was applied to detect mitochondrial distribution in oocytes. The MII oocytes were collected and moved to the pre-warmed MitoTracker solution with Hoechst 33,342 for 30 min at 37 ◦C with 5% CO_2_ in the air, protected from light. After washing three times with M2 medium, the oocytes were transferred to M2 medium on confocal dishes and observed under a laser scanning confocal microscope (SP8, Laica, Germany).

To measure fluorescence intensity, signals from both control and treatment oocytes were obtained using identical immunostaining procedures and confocal microscope parameters. Regions of interest (ROI) were defined using ImageJ (NIH, Bethesda, MD, USA), and the mean fluorescence intensity per unit area within the ROI was calculated. The mean values from all measurements were employed to compare the final average intensities between the control and treatment groups.

## In situ hybridization to study abundance of poly(A)-mRNA

Oocytes were fixed, permeabilized and blocked as previously described. Assessment of polyadenylated mRNAs was performed by in situ hybridization with single strand 5’-FITC-oligonucleotide(dT)_20_ probe (Sangon) in fixed oocytes (4% w/v PFA), 60 min at room temperature (RT); permeabilization in 0.5% Triton X-100 for 15 min at RT, hybridization with 1 mM single strand 5’-FITC-oligonukleotide(dT)_20_ probe for 1 h at 42°C, followed by 0.1% w/v BSA, 0.01% w/v Tween-20, 60 min at RT).

## In vitro transcription and preparation of mRNAs for microinjection

The cDNA for Dcp1a was subcloned into T7-driven vectors. All sequences were validated by Sanger sequencing before use (Sangon). To prepare mRNAs for microinjection, the expression vectors were linearized and in vitro transcribed using T7 mMESSAGE mMACHINE Ultra Kit (Life Technologies, AM1344), according to the manufacturer’s manual. Poly(A) tails (~ 200—250 bp) were added to transcribed mRNAs using the Poly(A) tailing kit (Life Technologies, AM1350) and were recovered by the MEGAclear Kit (Life Technologies, AM1908) and resuspended in nuclease-free water.c

## Microinjection

mRNAs or siRNAs were injected into cytoplasm of MII oocytes using a Narishige microinjector (Japan). Each oocyte was injected with 5–10 pL samples. The concentration of mRNAs was adjusted to 200 ng/µL and the siRNAs used were 10 μM. siRNAs targeting Dcp1a and the negative control were designed and synthesized by GenePharma (Shanghai, China) (Table S1). After injection, oocytes were washed and cultured in CZB medium at 37 °C with 5% CO_2_ in the air.

## RNA isolation and reverse transcription

Total RNA was extracted from 50 oocytes using the Arcturus PicoPure RNA Isolation Kit (Applied Biosystems, 12,204–01) according to the manufacturer’s manual, followed by reverse transcription to cDNA using the PrimeScript RT Master Mix (Takara, RR036A).

## Quantitative real time PCR (qRT-PCR)

qRT-PCR was performed with the CFX96 Real-Time PCR Detection System (Rio-rad) using TB Green Premix Ex Taq (Takara, RR420A). The data were calculated using the 2^−ΔΔCt^ method with normalization to β-actin. All primers were provided by Sangon Biotech (Shanghai, China) and are listed in Table S1. After incubation for 5 min at 95 °C, cDNAs were amplified using 45 cycles of 5 s at 95 °C, 30 s at 60 °C and 15 s at 72 °C. Each sample was measured in triplicate.

## mtDNA copy number analysis

Using the MALBAC single-cell WGA (Takara) method, we amplified the total DNA from five oocytes according to the manufacturer's protocol. Then, we directly used the amplified DNA for qRT-PCR with mtDNA-specific primers ND5 (Table S1). To normalize the mtDNA data, we compared them to the β-actin results (Table S1). Subsequently, qRT-PCR was performed as described in the qRT-PCR methods subsection.

## Extension poly(A) test

To measure poly(A) tail length in oocytes, extension poly(A) test (ePAT) was performed according to Amrei J. et al. [32]. Total RNA of 200 oocytes was denatured in the presence of 1 μL 100 mM PAT-anchor primer (Table S1) at 80 °C for 5 min. Then RT-master mix (4 μL 5 × Superscript III Buffer, Life Technologies; 1 μL 100 mM DTT, Life Technologies; 4 μL 2.5 mM dNTPs, Life Technologies; 1 μL RNaseOUT, Life Technologies; 5 U Klenow polymerase, New England Biolabs) was added and the samples were incubated at 25 °C for 1 h followed by inactivation at 80 °C for 10 min and cooling down to 55°C for 1 min. Subsequently, reverse transcription was performed by adding 1 μL Superscript III (Life Technologies), incubation at 55°C for 1 h and inactivation at 80°C for 10 min. PCR was conducted using Taq PCR Reaction Mix (Takara) under the following conditions: initial denaturation at 93°C for 5 min, amplification by 35 cycles of 30 s at 93°C, 60 s at 60°C, 60 s at 72°C, and 72°C for 10 min. The ePAT-anchor primer and the following gene-specific primers were used in Table S1. Extension PAT-fragments with variable poly(A) tail lengths were detected on a 2% agarose gel.

## Western blot

Oocytes were lysed with in the radioimmunoprecipitation assay (RIPA, Beyotime, Shanghai, China) lysis buffer with Phenylmethanesulfonyl fluoride (PMSF, Beyotime) and heated for 5 min at 95°C. Total oocyte proteins were separated on SDS-PAGE with a 5% stacking gel (10 ml; 6.9 ml ddHO, 1.3 ml 1M pH 8.8 Tris–HCl, 1.7 ml 30% acrylamide (acryl:bis acryl = 29:1), 100 μl 10% SDS, 100 μl 10% ammonium persulfate, and 10μl TEMED) and a 12% separating gel (10 ml; 3.3 ml ddHO, 2.5 ml 1.5 M pH 8.8 Tris–HCl, 4.1 ml 30% acrylamide (acryl:bis acryl = 29:1), 100 μl 10% SDS, 100 ul 10% ammonium persulfate, and 4 μl TEMED) at 120 V for 1.5 h and then electrophoretically transferred to Polyvinylidene fluoride (PVDF) membranes (Millipore). Membranes were blocked in TBST containing 5% defatted milk (Biorad) for 30 min. After probing with primary antibodies (anti-MOS antibody, Abcam, ab171937, 1:1000 dilution; anti-β-ACTIN antibody, GeneTex, GTX109639, 1:2000 dilution; anti-p-MAPK3/1 antibody, Cell Signaling Technology, 4370S, 1:2000 dilution; anti-MAPK3/1 antibody, Cell Signaling Technology, 4695S, 1:2000 dilution; anti-DCP1A antibody, Abcam, ab183709, 1:1000 dilution), the membranes were washed in TBST, incubated with an HRP-linked secondary antibody (HRP-conjugated anti-rabbit IgG, Cell Signaling Technology, 7074S, 1:2000 dilution; HRP-conjugated anti-mouse IgG, Cell Signaling Technology, 7076S, 1:3000 dilution) for 1 h, followed by three washes with TBST. Chemiluminescence was performed with ECL Plus (Servicebio, Wuhan, China) and signals were captured by Protein Simple imaging system.

## Quantitative proteomics analysis and MS data Analysis

About 750 oocytes per group were used in the TMT-based quantitative MS assay and lysed with RIPA buffer. The TMTsixplex Isobaric Label Reagent Set (catalog number 90061) from Thermo Fisher Scientific was used for the MS assay, which was performed on a Q-Exactive HF mass spectrometer equipped with a Nanospray Flex source. Samples were separated using a C18 column (15 cm × 75 µm) on an EASY-nLCTM 1200 system with a flow rate of 300 nL/min and a linear gradient of 75 min (0 ~ 63 min, 5–45% B; 63 ~ 65 min, 45–90% B; 65 ~ 75 min, 90%B; mobile phase A = 0.1% FA in water and B = 0.1% FA in ACN). Mass spectra in the range of 350–1500 m/z were obtained with a resolution of 60,000 and an AGC target value of 3e6 in full MS scans. The 20 most intense peaks in MS were subjected to higher-energy collisional dissociation (HCD) with a collision energy of 32, and MS/MS spectra were obtained with a resolution of 45,000 with an AGC target of 2e5 and a max injection time of 80 ms. The Q Exactive HF dynamic exclusion was set for 30.0 s, and the MS assay was run using positive mode. The raw data was searched against the sample protein database using ProteomeDiscoverer (v.2.4). The database search was carried out with Trypsin digestion specificity, and the presence of Alkylation on cysteine was taken into account as a fixed modification in the database searching. For protein quantification, a labeling method was utilized. To ensure accuracy, a global false discovery rate (FDR) of 0.01 was established, and protein groups considered for quantification needed a minimum of 2 peptides. Different expressed proteins were identified using the *t*-test function in python, proteins with foldchange > 1.2 or foldchange < 0.8333 and *P*-value < 0.05 were identified as Different expressed proteins. Gene Ontology (GO) enrichment analysis was carried out using Metascape, with a *P*-value < 0.05 [33].

## RNA-sequencing and RNA-seq data processing

RNA sequencing (RNA-seq) libraries (three replicates/group) were prepared as previously described [34]. Oocytes were washed three times with 0.1% PBS-PVA.

solution to avoid possible contamination; we used the Phusion Hot Start II High-Fidelity PCR Master Mix (F-565S, Thermo Fisher) to prepare libraries according to the instructions of the manufacturer of the NEBNext Ultra II DNA Library Prep Kit (E7805S, New England Biolabs, MA, USA). The libraries were sequenced on a NovaSeq 6000 platform (Illumina, CA, USA) according to the instructions of the manufacturer. To analyze RNA-seq data, we used Fastp to remove adapter sequences and low-quality bases from the raw sequence reads. Trimed reads were aligned to the mouse reference genome (mm10) using HISAT2 (version 2.1.0) [35]. Read count of genes were then calculated using FeatureCounts (version 2.0.1) [36] based on the GRCm38.101 annotation file which was downloaded from the Ensembl database. For each gene we normalized it’s reads count by Mitochondrial mRNA using the following methods: 1,000,000*gene_fragment_counts/mito_mRNA_read_counts.

## Statistical analyses

Data are presented as mean ± standard error of the mean (SEM), unless otherwise stated. The normality of the variable distribution was checked with the Shapiro–Wilk test. Comparisons between two groups were performed by two-tailed Student’s *t*-test, multiple comparisons among more than two groups were analyzed by one‐way ANOVA test using GraphPad Prism version 8 (GraphPad Software, San Diego, CA, USA). Differences were considered significant at *P* < 0.05.

## Results

### Cordycepin reduces fragmentation rate and improves developmental competence of postovulatory aging oocytes

**Fig. 1:**
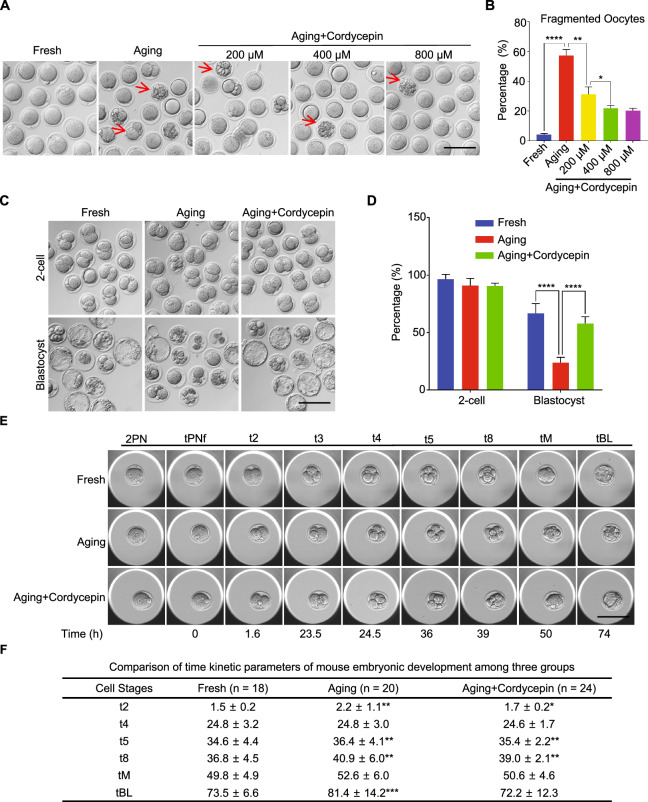
Effect of cordycepin supplementation on the integrity and developmental potential of postovulatory aging oocytes. A Representative images of fragmented oocytes of fresh, aging and cordycepin-treated groups. Red arrows: fragmented oocytes. Scale bar, 100 μm. **B** The rate of fragmentation was recorded in fresh (n = 123), aging (n = 167) and cordycepin-treated (n = 433) oocytes. Cordycepin was supplemented to the culture medium with concentrations at 200 μM (n = 143), 400 μM (n = 142) or 800 μM (n = 148), respectively. **C** Representative images of 2-cell embryos and blastocysts from fresh, aging and cordycepin-treated groups. Scale bar, 100 μm. **D** The rate of 2-cell embryos and blastocysts were recorded in fresh (n = 122), aging (n = 169) and cordycepin-treated (n = 179) groups. **E** Representative time-lapse images of fresh (n = 123), aging (n = 167) and cordycepin-treated (n = 433) embryos at the indicated stages. 2PN, two pronuclei; tPNf, time to pronuclei fading; t2, t3, t4, t5, and t8, time in hours post-tPNf for the embryo to reach the 2-, 3-, 4-, 5-, and 8-cell stages, respectively; tM, time for compaction; tB, time for the blastocoel to reach greater than or equal to half the volume of the embryo. Scale bar, 200 μm.** F** Comparison of time kinetic parameters of embryonic development among fresh (n = 18), aging (n = 20) and cordycepin-treated (n = 24) groups. Data of (**B**) and (**D**) were presented as mean percentage (mean ± SEM) from four independent experiments, and data of (F) ware from three independent experiments. Number of mice used in (**B**), (**D**) and (**F**) were 32, 20, and 9, respectively. Statistical analysis were performed with one-way ANOVA with Tukey's post hoc test. *P < 0.05, **P < 0.01, ***P < 0.001, ****P < 0.0001

To investigate the potential role of cordycepin in enhancing the quality of postovulatory aging oocytes, the integrity of ovulated mouse oocytes aging for 24 h in vitro, with or without cordycepin supplementation was evaluated. The majority of oocytes in the fresh group exhibited normal morphologies, while the aging oocytes displayed a higher degree of fragmentation (Fig. 1A). Quantitative analysis revealed a significant increase in the fragmentation rate of oocytes in aging group compared to fresh group (Fig. 1B). When oocytes were cultured with increasing concentrations of cordycepin (200, 400, and 800 μM), fragmentation rate was significantly reduced and notably cordycepin at 400 μM showed best effect (Fig. 1B). Thus, cordycepin at 400 μM was utilized in subsequent experiments. In both parthenogenetic activation and intracytoplasmic sperm injection (ICSI) experiments, we demonstrated that cordycepin supplementation improved the developmental competence of oocytes aging in vitro (Fig. 1C and 1D; Figure S1). Time-lapse monitoring showed that the interval from zygote to blastocyst was longer in aging oocytes compared to young oocytes; cordycepin significantly reduced the time to blastocyst formation in aging oocytes (Fig. 1E and 1F). Collectively, these finding suggest that cordycepin can protect postovulatory aging oocytes from fragmentation and improve their developmental competence.

## Cordycepin improves the spindle/chromosome morphology, mitochondrial distribution and reduces AMPK activation in oocytes aging in vitro

**Fig. 2:**
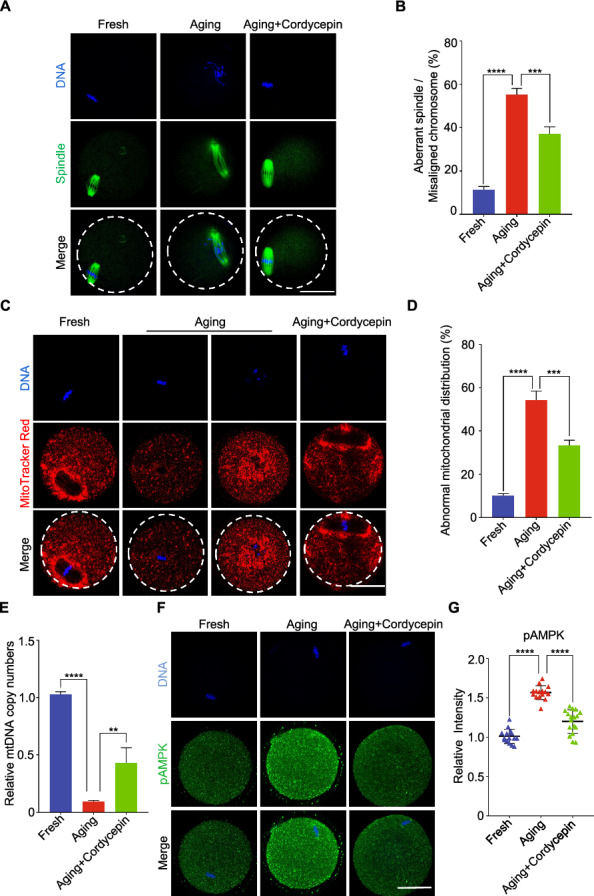
Effect of cordycepin on the spindle/chromosome morphology, mitochondrial distribution and AMPK activation in aging oocytes. **A** Representative images of spindle morphologies and chromosome alignment in fresh, aging and cordycepin-treated oocytes. Oocytes were immunostained with α-tubulin-FITC antibody to visualize the spindles and counterstained with Hoechst to visualize the chromosomes. Scale bar, 50 μm. **B** The rate of aberrant spindle with misaligned chromosomes was recorded in fresh (n = 61), aging (n = 42) and cordycepin-treated (n = 40) oocytes. **C** Representative images of the mitochondrial distribution in fresh, aging, and cordycepin-treated oocytes. Scale bar, 50 μm. **D** The rates of abnormal distribution of mitochondria in fresh (n = 42), aging (n = 33) and cordycepin-treated (n = 39) oocytes. **E** mtDNA copy numbers of fresh (n = 15), aging (n = 15), and cordycepin-treated (n = 15) oocytes. **F** Representative images of pAMPK in fresh, aging, and cordycepin-treated oocytes. Scale bar, 50 μm. **(G)** Fluorescence intensity of pAMPK signals was measured in fresh (n = 15), aging (n = 15) and cordycepin-treated (n = 17) oocytes. Data of (**B**), (**D**), (**E**) and **G** were presented as mean percentage (mean ± SEM) from three independent experiments. In (**B**), (**D**) and (**G**), a total of 15 mice were used; In (**E**), 6 mice were used. Statistical analysis were performed with one-way ANOVA with Tukey's post hoc test. **P < 0.01, ***P < 0.001, ****P < 0.0001

Spindle integrity is essential for accurate chromosome alignment and serves as a crucial indicator of oocyte quality. Consequently, we investigated spindle/chromosome organization in aging oocytes. In fresh oocytes, we observed a typical barrel-shape spindle with well-aligned chromosomes (Fig. 2A). Immunostaining images revealed a range of disorganized spindle apparatuses with misaligned chromosomes in aging oocytes. Quantitative analysis indicated that the percentage of spindle/chromosome defects was increased in aging oocytes but reduced after cordycepin supplementation (Fig. 2A-2B). Mitochondria serve as another indicator of oocyte quality. Thus, we examined mitochondrial distribution in oocytes. In fresh oocytes, mitochondria congregated around chromosomes and were homogeneously distributed throughout the cytoplasm. In contrast, aging oocytes exhibited a strikingly increased proportion of mitochondria that entirely or partially lost their normal distribution around chromosomes and displayed an aggregated distribution pattern in the cytoplasm. Quantitative analysis revealed that more than 54% of aging oocytes displayed this abnormal distribution, whereas cordycepin supplementation reduced the occurrence to 33% (Fig. 2C and 2D). Furthermore, we observed that cordycepin rescued the reduction in mtDNA copy number in aging oocytes compared to controls (Fig. 2E).

A previous study has demonstrated that the AMP-activated protein kinase (AMPK) pathway is activated in postovulatory aging oocyte [10]. Therefore, we evaluated the effect of cordycepin on AMPK activation in aging oocytes and found that it significantly decreased the level of active AMPK induced by postovulatory aging (Fig. 2F and 2G). These observations suggest that cordycepin supplementation could partially rescue the aging-induced defects in spindle/chromosomes organization, mitochondrial distribution and reduce AMPK activation.

## Identification of target effectors of cordycepin in aging oocytes by proteomics analysis

**Fig. 3:**
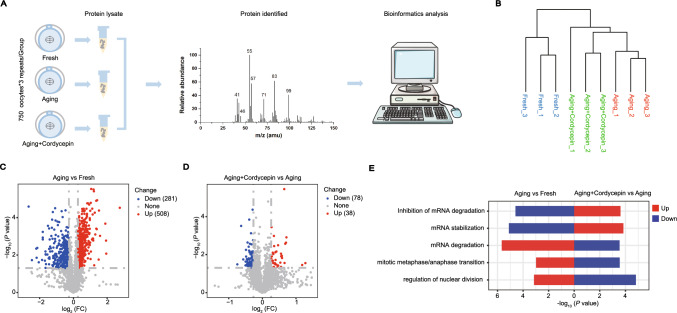
Proteomics analysis of cordycepin-treated oocytes compared to aging oocytes. **A** Schematic diagram of the proteomics analysis. **B** Unsupervised clustering result of samples based on proteomics data. **C** Volcano plot of the DEPs in aging compared to fresh oocytes (downregulated, blue; upregulated, red). **D** Volcano plot of the DEPs in cordycepin-treated oocytescompared to aging oocytes (downregulated, blue; upregulated, red). **(E)** gene ontology (*GO*) analysis of the two sets of DEPs

To explore the underlying mechanisms by which cordycepin improves the quality of aging oocytes, we conducted a proteomics analysis (Fig. 3A). Unsupervised hierarchical clustering revealed high intragroup consistency and effectively differentiated the fresh, aging, and cordycepin groups (Fig. 3B), indicating distinct protein expression patterns across these three groups. A total of 789 differentially expressed proteins (DEPs) were identified between aging and fresh oocytes, including 508 upregulated and 281 downregulated proteins in aging oocytes (Fig. 3C; Table S2). In addition, cordycepin supplementation resulted in 38 upregulated and 78 downregulated proteins compared to the aging oocytes (Fig. 3D; Table S3). Gene Ontology (GO) analysis of DEPs revealed that proteins enriched in cell cycle-related pathways exhibited upregulation in aging oocytes compared to fresh group, but displayed downregulation in cordycepin-treated oocytes (Fig. 3E). Intriguingly, our findings indicated that mRNA stabilization was markedly impacted during postovulatory aging, including the activation of mRNA degradation. However, this degradation was effectively inhibited by cordycepin supplementation (Fig. 3E).

## Cordycepin prevents degradation of maternal proteins and mRNAs in aging oocytes

**Fig. 4:**
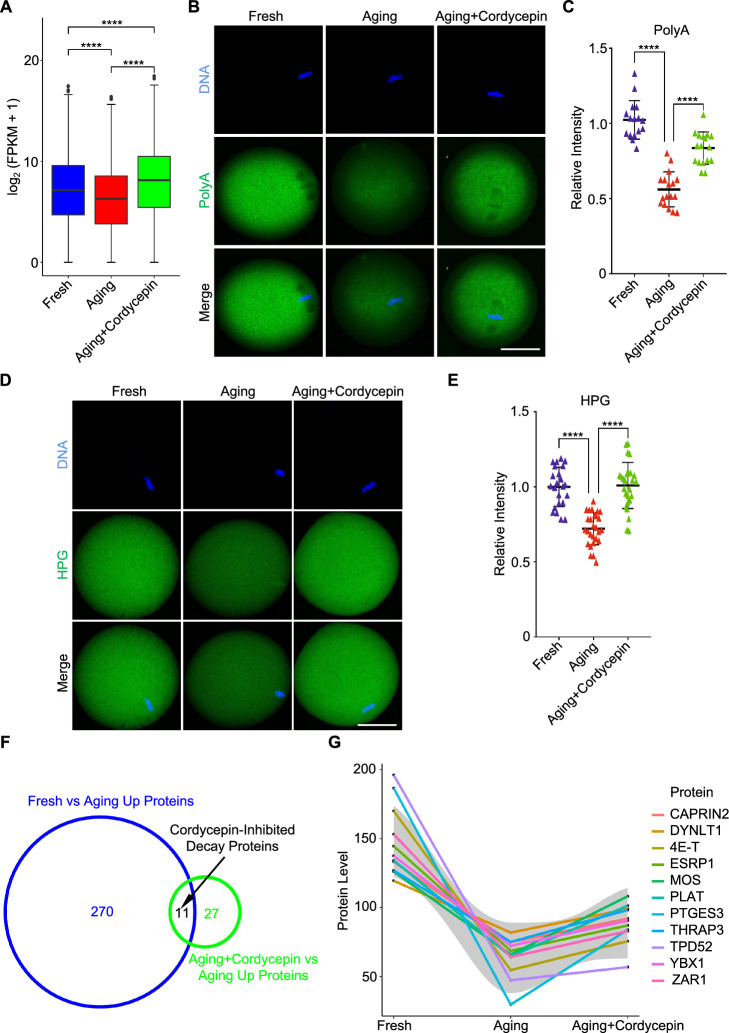
Cordycepin inhibits the degradation of maternal proteins and mRNAs in aging oocytes. **A** Box plot showing the polyadenylated mRNA levels in fresh, aging, and cordycepin-treated oocytes through RNA-Seq. The box indicates the upper and lower quantiles, the thick line in the box indicates the median and whiskers indicates 2.5th and 97.5th percentiles. **B** Representative images of polyadenylated mRNA in fresh, aging, and cordycepin-treated oocytes. Scale bar, 50 μm. **C** Fluorescence intensity of polyadenylated mRNA signals was measured in fresh (n = 16), aging (n = 17) and cordycepin-treated (n = 17) oocytes. **D** HPG fluorescent staining showing the protein synthesis activity in fresh, aging, and cordycepin-treated oocytes. Scale bar, 50 μm. **E** Fluorescence intensity of HPG signals was measured in fresh (n = 23), aging (n = 27) and cordycepin-treated (n = 26) oocytes. **F** Venn diagrams showing the overlap in proteins that were significantly degraded during postovulatory aging (fold change (fresh/aging) > 1.2) and upregulated in this process when cordycepin supplemented (fold change (cordycepin-treated/aging) > 1.2). **G** 11 maternal proteins that were significantly inhibited from degradation by cordycepin during postovulatory aging. Data of (A), (C) and (E) were presented as mean percentage (mean ± SEM) from three independent experiments. In (**C**) and (**E**), a total of 9 mice were used. Statistical analysis were performed with one-way ANOVA with Tukey's post hoc test. ****P < 0.0001

To investigate the effect of cordycepin on preservation of maternal mRNA stability in aging oocytes, we performed RNA sequencing (RNA-Seq). Inhibition of total mRNA decay was observed when cordycepin was supplemented during aging (Fig. 4A), which was verified by immunofluorescence using poly(dT)_20_-oligonucleotides (Fig. 4B and 4C). Global translation activity was assessed using homopropargylglycine (HPG), a methionine analogue. HPG signals decreased during postovulatory aging but were restored by cordycepin (Fig. 4D and 4E). Then, our focus was directed towards the examination of upregulated proteins in oocytes treated with cordycepin. We identified 11 maternal proteins that were significantly inhibited from degradation by cordycepin during postovulatory aging. These included several important maternal effect proteins such as ZAR1 [18], YBX1 [37, 38] and MOS [39], in conjunction with mRNA-binding proteins like 4E-T [40], CAPRIN2 [41], ESRP1 [42] and THRAP3 [43] (Fig. 4F and 4G). Herein, the gene expression patterns corresponding to the 11 cordycepin-inhibited decay proteins was similar to the proteomic results (Figure S2A and S2B).

The moloney sarcoma oncogene (MOS), one of the cordycepin-inhibited decay proteins, plays crucial roles in the maintaining MII arrest and facilitating maternal-to-zygotic transition through the MOS-MAPK3/1 pathway [44]. Here, immunofluorescence and Western blot analyses showed that the MOS protein level declined during aging but restored by cordycepin supplementation (Figure S3). Next, we examined MAPK3/1 activation by staining for phosphorylated MAPK3/1 (p-MAPK3/1). The p-MAPK3/1 signal decreased after aging and was rescued by cordycepin (Figure S4A and S4B), which was also confirmed by Western blot (Figure S4C and S4D). To further investigate the involvement of MOS-MAPK3/1 in the effect of cordycepin on improving aging oocyte quality, a MAPK3/1 specific inhibitor (U0126) was employed in aging oocytes. Cordycepin was only able to restore the p-MAPK3/1 level in the presence of 1 μM, but not 3 μM of U0126 (Figure S4E and S4F). Furthermore, we discovered that cordycepin partially reduces fragmentation rate and improves developmental competence of aging oocytes in the presence of 1 μM, but not 3 μM, of U0126 (Figure S4G and S4H). In summary, these findings indicate that cordycepin can effectively prevent the degradation of maternal proteins and mRNAs induced by postovulatory aging.

## Cordycepin-mediated inhibition of maternal mRNAs degradation is achieved through the DCP1A polyadenylation suppression

**Fig. 5:**
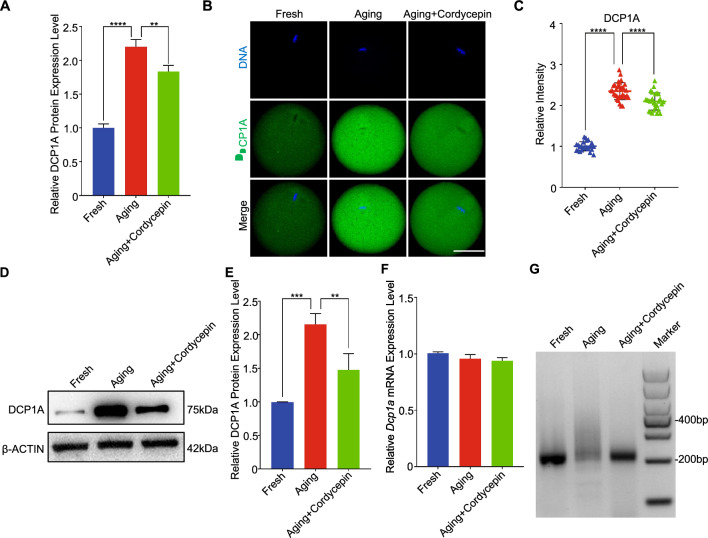
Cordycepin inhibits the translation activities of DCP1A through the polyadenylation suppression. **A** Protein levels of DCP1A examined by proteomics in fresh, aging, and cordycepin-treated oocytes. **B** Representative images of DCP1A in fresh, aging, and cordycepin-treated oocytes. Scale bar, 50 μm. **C** Fluorescence intensity of DCP1A signals was measured in fresh (n = 24), aging (n = 33) and cordycepin-treated (n = 27) oocytes. **D** Protein levels of DCP1A examined by western blot analysis in fresh, aging, and cordycepin-treated oocytes. 200 oocytes for each sample were collected and immunoblotted for DCP1A and β-ACTIN. **E** Quantitative analysis of DCP1A protein levels by Western blot. **F** Quantitative RT-PCR results showing the expression level of the Dcp1a gene in fresh, aging, and cordycepin-treated oocytes. **G** Changes in poly(A)-tail length of Dcp1a mRNA in fresh, aging, and cordycepin-treated oocytes. Data of (**C**), (**E**) and (**F**) were presented as mean percentage (mean ± SEM) from three independent experiments. Number of mice used in (**C**), (**D**) and (**F**) were 6, 90, and 24, respectively. Statistical analysis were performed with one-way ANOVA with Tukey's post hoc test. **P < 0.01, ***P < 0.001, ****P < 0.0001

The decapping complex plays an essential role in the degradation of maternal mRNAs during oocyte maturation. Intriguingly, proteomic results showed that DCP1A, a key protein in the decapping complex, exhibits a marked elevation during postovulatory aging, but was inhibited by cordycepin (Fig. 5A). This trend of DCP1A levels across the three groups was further verified by both immunofluorescence and Western blot analyses (Fig. 5B-5E). In contrast to the protein levels, the relative transcript levels of DCP1A were not changed (Fig. 5F). Therefore, we postulated that the alterations in DCP1A protein level was modulated via cytoplasmic polyadenylation. To assess the length of the poly(A) tail of DCP1A mRNA, we performed an extension poly(A) test (ePAT). As expected, we observed a lengthening of the poly(A) tail for DCP1A mRNA in aging oocytes compared to controls, while this lengthening was inhibited by cordycepin (Fig. 5G). These observations indicate that cordycepin decreases the protein level of DCP1A via polyadenylation suppression.

**Fig. 6:**
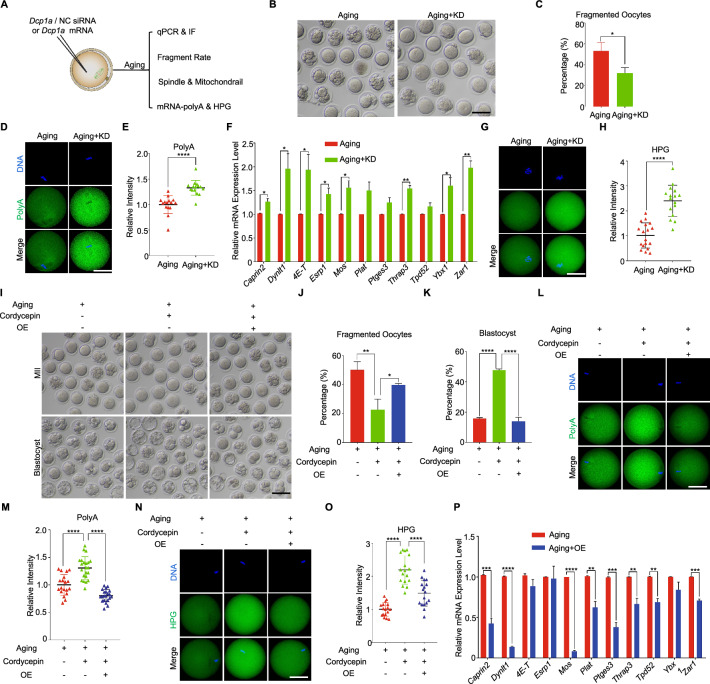
Cordycepin inhibits maternal mRNAs degradation through suppressing the elevation of DCP1A protein. **A** Schematic presentation of the Dcp1a knockdown (KD) and overexpression (OE) experimental protocol. **B** Representative images of fragmented oocytes in aging and Dcp1a knockdown groups. Scale bar, 100 μm. **C** The rate of fragmentation was recorded in aging (n = 122) and the DCP1A-KD aging (n = 127) oocytes. **D** Representative images of polyadenylated mRNA in aging and DCP1A-KD aging oocytes. Scale bar, 50 μm. **(E)** Fluorescence intensity of polyadenylated mRNA signals was measured in aging (n = 15) and DCP1A-KD aging (n = 15) oocytes.** F** Quantitative RT-PCR results showing the expression level of the 11 maternal genes in aging and DCP1A-KD aging oocytes. **G** HPG fluorescent staining showing the protein synthesis activity in aging and DCP1A-KD aging oocytes. Scale bar, 50 μm. **H** Fluorescence intensity of HPG signals was measured in aging (n = 18) and DCP1A-KD aging (n = 16) oocytes. **I** Representative images of fragmented oocytes and blastocysts in aging, cordycepin and cordycepin + DCP1A-OE groups. Scale bar, 100 μm.** J** The rate of fragmentation was recorded in aging (n = 94), cordycepin (n = 116) and cordycepin + DCP1A-OE (n = 83) oocytes. **K** The rate of blastocysts was recorded in aging (n = 118), cordycepin (n = 120) and the cordycepin + DCP1A-OE (n = 127) groups.** L** Representative images of polyadenylated mRNA in aging, cordycepin and cordycepin + DCP1A-OE oocytes. Scale bar, 50 μm. **M** Fluorescence intensity of polyadenylated mRNA signals was measured in aging (n = 19), cordycepin (n = 22) and cordycepin + DCP1A-OE (n = 21) oocytes. **N** HPG fluorescent staining showing the protein synthesis activity in aging, cordycepin and cordycepin + DCP1A-OE oocytes. Scale bar, 50 μm. **O** Fluorescence intensity of HPG signals was measured in aging (n = 17), cordycepin (n = 17) and cordycepin + DCP1A-OE (n = 17) oocytes. **P** Quantitative RT-PCR results showing the expression level of the 11 maternal genes in aging and DCP1A-OE aging oocytes. Data of (**C**), (**E**), (**F**), (**H**), (**J**), (**K**), (**M**), (**O**) and (**P**) were presented as mean percentage (mean ± SEM) from three independent experiments. Number of mice used in (**C**), (**E**) & (**H**), (**F**), (**J**) & (**K**), (**M**) & (**O**) and (**P**) were 12, 9, 18, 27, 12 and 18, respectively. In (C), (**E**), (**F**), (**H**) and (**P**), statistical analysis were performed with Student's t test (two-tailed); and in (**J**), (**K**), (**M**) and (**O**) one-way ANOVA with Tukey's post hoc test was used for statistical analysis. *P < 0.05, **P < 0.01, ***P < 0.001, ****P < 0.0001

To further substantiate the notion that cordycepin inhibits maternal mRNAs degradation through suppressing the elevation of DCP1A protein, we performed DCP1A knockdown (KD) and overexpression (OE) (Fig. 6A). The effectiveness of DCP1A KD or DCP1A OE was confirmed through qPCR and immunofluorescence (Figure S5). It was observed that DCP1A KD significantly decreased the rates of fragmentation, spindle anomalies and abnormal mitochondrial distribution (Fig. 6B and 6C; Figure S6A-6E). Concurrently, the total mRNA abundance (Fig. 6D and 6E), along with the mRNA levels of 11 cordycepin-inhibited decay maternal genes, exhibited a notable increase in KD-aging oocytes (Fig. 6F). Furthermore, the HPG signals displayed a markedly elevation in DCP1A-KD aging oocytes compared to the aging oocytes (Fig. 6G and 6H). Upon DCP1A overexpression, we demonstrated that the beneficial effects of cordycepin on reducing fragmentation, spindle anomalies, abnormal mitochondrial distribution, and improving developmental competence of aging oocytes were inhibited (Fig. 6I-K; Figure S6F-I). Overexpression of DCP1A also prevented the cordycepin-induced increase in total mRNA abundance and HPG signals in aging oocytes (Fig. 6L-6O). Furthermore, the expression levels of these cordycepin-inhibited decay maternal genes were decreased in DCP1A-OE aging oocytes compared to the aging oocytes (Fig. 6P). These results suggest that cordycepin suppresses the elevation of DCP1A protein by inhibiting polyadenylation during postovulatory aging, subsequently impeding the degradation of maternal mRNAs and ultimately enhancing the quality of aging oocytes.

## Cordycepin inhibits the elevation of DCP1A and the degradation of maternal mRNAs in human postovulatory aging oocytes

**Fig. 7:**
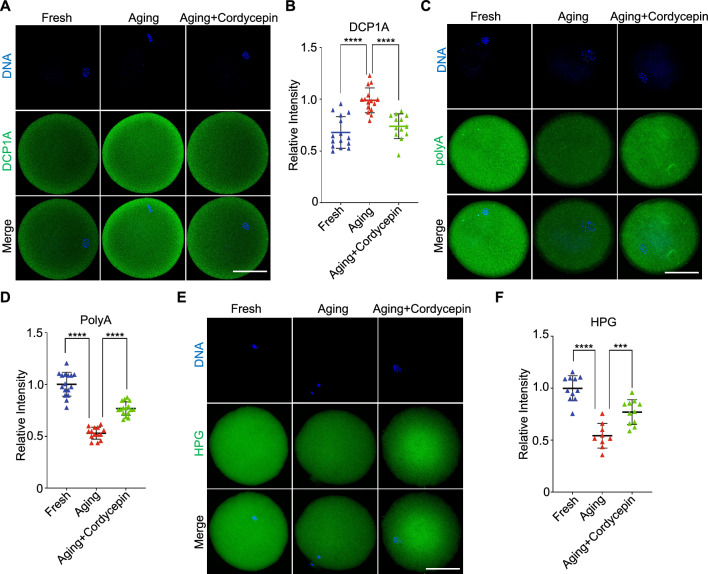
Cordycepin inhibits the elevation of DCP1A and the degradation of maternal mRNAs in human aging oocytes*.* **A** Representative images of DCP1A in human fresh, aging, and cordycepin-treated oocytes. Scale bar, 50 μm. **B** Fluorescence intensity of DCP1A signals was measured in human fresh (n = 15), aging (n = 15) and cordycepin-treated (n = 13) oocytes.** C** Representative images of polyadenylated mRNA in human fresh, aging, and cordycepin-treated oocytes. Scale bar, 50 μm. **D** Fluorescence intensity of polyadenylated mRNA signals was measured in human fresh (n = 16), aging (n = 14) and cordycepin-treated (n = 16) oocytes. **E** HPG fluorescent staining showing the protein synthesis activity in human fresh, aging, and cordycepin-treated oocytes. Scale bar, 50 μm. **F** Fluorescence intensity of HPG signals was measured in human fresh (n = 11), aging (n = 9) and cordycepin-treated (n = 11) oocytes. Data of (**B**), (**D**) and (**F**) were presented as mean percentage (mean ± SEM) from three independent experiments. Number of patients used in (**B**), (**D**) and (**F**) were 18, 21 and 15, respectively. Statistical analysis were performed with one-way ANOVA with Tukey's post hoc test. ***P < 0.001, ****P < 0.0001

The observation that cordycepin reduces DCP1A protein level and inhibits maternal mRNAs degradation prompted us to investigate the potential existence of a similar mechanism in human postovulatory aging oocytes. We performed immunostaining of DCP1A in human aging oocytes and found that, similar to results observed in mice, DCP1A signal increased during postovulatory aging but was inhibited by cordycepin (Fig. 7A and 7B). Furthermore, total mRNA abundance and HPG signals were markedly reduced in human aging oocytes relative to fresh oocytes, while displaying comparable levels between cordycepin-treated oocytes and fresh oocytes (Fig. 7C-7F). Therefore, the mechanism of cordycepin-mediated inhibition of maternal mRNAs degradation during postovulatory aging may be conserved between mice and human.

## Discussion

Postovulatory aging, starting from ovulation and progressing continuously, is a significant factor contributing to the impairment of oocyte quality and the failure of human ART procedures, especially when rescue ICSI were employed [45, 46]. Although several drugs such as melatonin and coenzyme Q10 have shown the ability in delaying postovulatory aging, they are not used clinically and the precise molecular mechanisms governing the aging pathway remain insufficiently elucidated [47–50]. In this study, we demonstrate the effectiveness of cordycepin in improving the quality of postovulatory aging oocytes by primarily inhibiting the degradation of maternal proteins and mRNAs.

Maternal proteins are essential for acquisition and maintenance of oocyte developmental competence, affecting a variety of events such as oocyte maturation, fertilization, OET, zygotic gene activation (ZGA), and embryonic development [17, 18, 51, 52]. Recent findings reveal that postovulatory aging leads to reduction in the abundance of several maternal proteins, including BRG1 and MATER [7]. Our proteomic analysis has identified a substantial downregulation of numerous proteins in aging oocytes. To explore the mechanism underlying the cordycepin-mediated effects on aging oocytes, we focused on proteins that decreased during aging, but restored following cordycepin supplementation. These were then referred to as “cordycepin-inhibited decay proteins”. MOS, one of cordycepin-inhibited decay proteins, activates the MAPK3/1 signaling cascade during oocyte maturation. Deficiency of MOS or MAPK3/1 in mice and human results in oocyte MII arrest failure, spindle abnormality, and early embryo developmental arrest [39, 53–55]. The findings from our investigation revealed that cordycepin supplementation increased levels of MOS and p-MAPK3/1 in aging oocytes. In addition, inhibition of p-MAPK3/1 suppressed the recovery of fragmentation and developmental competence by cordycepin, suggesting a possible partial involvement of MOS degradation inhibition in cordycepin-mediated restoration of quality in aging oocytes. Therefore, our findings suggest that cordycepin might improve the quality of aging oocytes through the inhibition of maternal proteins degradation.

Oocyte maturation instigates a shift from mRNA stability towards instability, leading to the degradation of thousands of diverse mRNAs [56]. In the absence of transcriptional activity during this process, the regulation of mRNAs degradation principally determines the abundance of corresponding proteins [31, 57]. Therefore, we speculated that cordycepin inhibits the degradation of maternal mRNAs during postovulatory aging. Here, immunofluorescence of poly(dT)_20_-oligonucleotides and HPG verified that total mRNA abundance and global translation activity decreased in aging oocytes, but restored after cordycepin supplementation. Recent studies have demonstrated the important role of mRNA-binding proteins in maintaining mRNAs stability. Here, several mRNA-binding proteins such as zygote arrest-1 (ZAR1), eIF4E-transporter protein (4E-T) and Y-box binding protein-1 (YBX1) were identified as cordycepin-inhibited decay proteins. ZAR1 is one of the first reported mammalian maternal effect genes; its knockout results in compromised translational activation of maternal mRNAs encoding important meiosis and OET factors [18, 58, 59]. 4E-T, a type of eIF4E-binding proteins (4E-BPs), are required to protect the deadenylated target mRNA from degradation by blocking decapping [40]. YBX1, a member of Y-box binding proteins, serves an important role in maternal mRNA degradation, alternative splicing, and transcriptional activity required for early embryonic development [37, 38, 60]. Consequently, these observed degradation of mRNA-binding proteins during aging could further accelerate mRNAs degradation. Despite the confirmed inhibition of maternal mRNAs degradation by cordycepin, the underlaying mechanisms remain elusive.

Cordycepin, an analogue of nucleoside, partakes in RNA synthesis. Due to the absence of a 3’-hydroxyl moiety, cordycepin incorporates into sites ordinarily occupied by nucleic acids, thereby inhibiting polyadenylation [61]. In eukaryotic cells, functional mRNA possesses a 5’-cap structure and a 3’-poly(A) tail that regulate translation and mRNA stability [62–64]. Hence, mRNAs degradation typically encompasses the deadenylation of the 3’- poly(A) tail and decapping of the 5’-cap [64, 65]. Given the properties of cordycepin in polyadenylation inhibition, we propose that it might suppress the elevation of certain proteins implicated in directing mRNA degradation, thereby inhibiting maternal mRNAs degradation during oocyte aging. We therefore screened for proteins displaying increased level in aging oocytes but inhibited by cordycepin. Notably, we identified DCP1A, a recognized protein responsible for mRNA decapping [63]. Previous study has demonstrated that DCP1A dramatically increases during oocytes maturation via cytoplasmic polyadenylation, a crucial process for proper maternal mRNAs degradation and OET [66]. We speculate that DCP1A mRNA is continuously being polyadenylated during postovulatory aging, leading to a rise in DCP1A protein and the abnormal degradation of maternal mRNAs. Our ePAT and protein level analyses verify the inhibition of polyadenylation and the increase of DCP1A by cordycepin during aging. In addition, DCP1A knockdown and overexpression revealed that cordycepin-mediated inhibition of maternal mRNAs degradation is executed via the suppression of DCP1A polyadenylation. Most notably, we also observed the restoration of DCP1A, global mRNA abundance, and translation activity by cordycepin in human aging oocytes. This suggests that the mechanism by which cordycepin enhances the quality of aging oocytes appears conserved among mammals.

## Conclusion

**Fig. 8:**
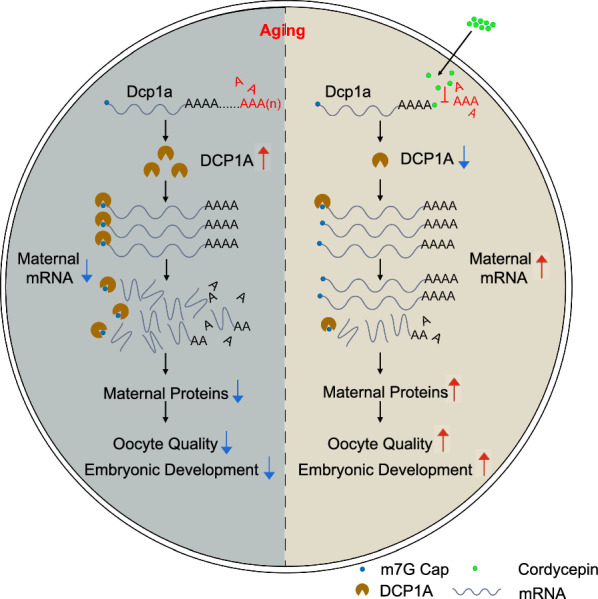
Schematic diagram of cordycepin delays postovulatory aging of mammalian oocytes through inhibition of maternal mRNAs degradation via DCP1A polyadenylation suppression

In summary, we demonstrate that cordycepin, a natural nucleoside analogue, prevents oocytes from aging by overcoming the abnormal maternal mRNA degradation, through suppressing the polyadenylation of DCP1A (Fig. 8). This finding may provide an effective approach to prevent postovulatory aging of oocytes in human ART. Future clinical investigations are needed to define the effects of cordycepin on human oocyte developmental competence.

### Supplementary Information

Below is the link to the electronic supplementary material.

Supplementary file1 Figure S1. Effect of cordycepin supplementation on the parthenogenetic embryonic developmental potential of postovulatory aging oocytes (A) Representative images of 2-cell embryos and blastocysts from fresh, aging and cordycepin-treated groups. Scale bar, 100 μm. (B) The rates of 2-cell embryos and blastocysts were recorded in fresh (n = 135), aging (n = 170) and cordycepin-treated (n = 176) groups. A total of 28 mice were used. Data was presented as mean percentage (mean ± SEM) from four independent experiments. Statistical analysis were performed with one-way ANOVA with Tukey's post hoc test. *P < 0.05, **P < 0.01 (PDF 1133 KB)

Supplementary file2 Table S1. Primer sequences used in this study (XLS 24 KB)

Supplementary file3 (XLS 298 KB)

Supplementary file4 Table S3. Differentially expressed proteins between cordycepin-treated and aging oocytes (XLS 59 KB)

Supplementary file5 Figure S2. Gene expression patterns to the corresponding 11 cordycepin-inhibited decay proteins (A) Transcript levels of the corresponding 11 cordycepin-inhibited decay proteins examined by RNA-seq in fresh, aging, and cordycepin-treated oocytes. (B) Quantitative RT-PCR results showing the expression level of the corresponding 11 cordycepin-inhibited decay proteins in fresh, aging, and cordycepin-treated oocytes. A total of 21 mice were used. Data was presented as mean percentage (mean ± SEM) from three independent experiments. Statistical analysis were performed with one-way ANOVA with Tukey's post hoc test. *P < 0.05, **P < 0.01, ***P < 0.001, ****P < 0.0001 (PDF 460 KB)

Supplementary file6 Figure S3. Cordycepin inhibits the degradation of MOS protein in aging oocytes (A) Representative images of MOS expression in fresh, aging, and cordycepin-treated oocytes. Scale bar, 50 μm. (B) Fluorescence intensity of MOS signals was measured in fresh (n = 15), aging (n = 14) and cordycepin-treated (n = 1¬¬7) oocytes. (C) Protein levels of MOS examined by Western blot analysis in fresh, aging, and cordycepin-treated oocytes. 200 oocytes for each group were collected and immunoblotted for MOS and β-ACTIN. (D) Quantitative analysis of MOS protein levels from western blot. Data of (B) and (D) were presented as mean percentage (mean ± SEM) from three independent experiments. Number of mice used in (B) and (C) were 6, and 75, respectively. Statistical analysis were performed with one-way ANOVA with Tukey's post hoc test. *P < 0.05, **P < 0.01, ****P < 0.0001. (PDF 468 KB)

Supplementary file7 Figure S4. Cordycepin improves the MAPK3/1 pathway in aging oocytes. (A) Representative images of p-MAPK3/1 expression in fresh, aging, and cordycepin-treated oocytes. Scale bar, 50 μm. (B) Fluorescence intensity of p-MAPK3/1 signals was measured in fresh (n = 13), aging (n = 14) and cordycepin-treated (n = 17) oocytes. (C) Protein levels of p-MAPK3/1 examined by Western blot analysis in fresh, aging, and cordycepin-treated oocytes. 200 oocytes for each group were collected and immunoblotted for p-MAPK3/1 and MAPK3/1. (D) Quantitative analysis of p-MAPK3/1 protein levels by Western blot. (E) Representative images of p-MAPK3/1 expression in aging, cordycepin-treated, U0126 (1 μM), U0126 (1 μM) + cordycepin, U0126 (3 μM) and U0126 (3 μM) + cordycepin oocytes. Scale bar, 50 μm. (F) p-MAPK3/1 fluorescence intensity in aging (n = 17), cordycepin (n = 22), U0126 (1 μM) (n = 18), U0126 (1 μM) + cordycepin (n = 17), U0126 (3 μM) (n = 14) and U0126 (3 μM) + cordycepin (n = 15) oocytes. (G) The rate of fragmentation was recorded in aging (n = 97), cordycepin (n = 92), U0126 (1 μM) (n = 73), U0126 (1 μM) + cordycepin (n = 117), U0126 (3 μM) (n = 75) and U0126 (3 μM) + cordycepin (n = 83) oocytes. (H) The rate of blastocysts was recorded in aging (n = 120), cordycepin (n = 91), U0126 (1 μM) (n = 113), U0126 (1 μM) + cordycepin (n = 120), U0126 (3 μM) (n = 106) and U0126 (3 μM) + cordycepin (n = 114) oocytes. Data of (B), (D), (F), (G) and (H) were presented as mean percentage (mean ± SEM) from three independent experiments. Number of mice used in (B), (D), (F), (G) and (H) were 6, 75, 9, 24, 24 and 27, respectively. Statistical analysis were performed with one-way ANOVA with Tukey's post hoc test. *P < 0.05, **P < 0.01, ***P < 0.001, ****P < 0.0001, ns: no significance (PDF 1065 KB)

Supplementary file8 Figure S5. Dcp1a knockdown and overexpression in oocytes. (A) Quantitative RT-PCR results showing the expression level of the Dcp1a gene in aging and DCP1A-KD aging oocytes. (B) Fluorescence intensity of DCP1A signals was measured in aging and DCP1A-KD aging oocytes. Scale bar, 50 μm. (C) Fluorescence intensity of DCP1A signals was measured in aging (n = 28) and DCP1A-KD aging (n = 21) oocytes. (D) Quantitative RT-PCR results showing the expression level of the Dcp1a gene in aging and DCP1A-OE aging oocytes. (E) Fluorescence intensity of DCP1A signals was measured in aging and DCP1A-OE aging oocytes. Scale bar, 50 μm. (F) Fluorescence intensity of DCP1A signals was measured in aging (n = 19) and DCP1A-OE aging (n = 19) oocytes. Data of (A), (C), (D) and (F) were presented as mean percentage (mean ± SEM) from three independent experiments. Number of mice used in (A), (C), (D) and (F) were 15, 6, 15 and 6, respectively. Statistical analysis were performed with Student's t test (two-tailed). ****P < 0.0001 (PDF 1777 KB)

Supplementary file9 Figure S6. Effect of DCP1A-KD and DCP1A-OE on the spindle assembly and mitochondrial distribution in aging oocytes. A Representative images of spindle morphologies in aging and DCP1A-KD aging oocytes. Oocytes were immunostained with α-tubulin-FITC antibody to visualize the spindles and counterstained with Hoechst to visualize the chromosomes. Scale bar, 50 μm. (B) The rate of aberrant spindle was recorded in aging (n = 45) and DCP1A-KD aging (n = 58) oocytes. (C) Representative images of the mitochondrial distribution in aging, and DCP1A-KD aging oocytes. Scale bar, 50 μm. (D) The rates of abnormal distribution of mitochondria in aging (n = 49) and DCP1A-KD aging (n = 49) oocytes. (E) mtDNA copy numbers of aging (n = 15), and DCP1A-KD aging (n = 15) oocytes. (F) Representative images of spindle morphologies in aging, cordycepin and cordycepin + DCP1A-OE oocytes. Oocytes were immunostained with α-tubulin-FITC antibody to visualize the spindles and counterstained with Hoechst to visualize the chromosomes. Scale bar, 50 μm. (G) The rate of aberrant spindle was recorded in aging (n = 42), cordycepin (n = 40) and cordycepin + Dcp1a-OE (n = 38) oocytes. (H) Representative images of the mitochondrial distribution in aging, cordycepin and cordycepin + DCP1A-OE oocytes. Scale bar, 50 μm. (I) The rates of abnormal distribution of mitochondria in aging (n = 44), cordycepin (n = 47) and cordycepin + DCP1A-OE (n = 48) oocytes. (J) mtDNA copy numbers of aging (n = 15), cordycepin (n = 15) and cordycepin + DCP1A-OE (n = 15) oocytes. Data of (B), (D), (E), (G), (I) and (J) were presented as mean percentage (mean ± SEM) from three independent experiments. Number of mice used in (B) & (D), (C), (G) & (I) and (J) were 9, 6, 15 and 6, respectively. In (B), (D) and (E), statistical analysis were performed with Student's t test (two-tailed); and in (G), (I) and (J) one-way ANOVA with Tukey's post hoc test was used for statistical analysis. *P < 0.05, **P < 0.01, ***P < 0.001, ****P < 0.0001 (PDF 912 KB)

## Data Availability

The mass spectrometry proteomics data have been deposited to the ProteomeXchange Consortium via the PRIDE [67] partner repository with the dataset identifier PXD042600. The raw RNAseq data for this study can be found in the NCBI public database at this URL link: https://www.ncbi.nlm.nih.gov/bioproject/PRJNA978320.

## Abbreviations

ART: Assisted reproductive technology
IVF: In vitro Fertilization
ICSI: Intracytoplasmic sperm injection
OET: Oocyte to embryo transition
ZGA: Zygotic genome activation
ICR: Institute of Cancer Research
PMSG: Pregnant mare serum gonadotropin
HCG: Human chorionic gonadotropin
GV: Germinal vesicle
MII: Metaphase II
IRB: Institutional Review Board
DMSO: Dimethyl sulfoxide
HPG: Homopropargylglycine
PFA: Paraformaldehyde
PBS: Phosphate-buffered saline
PVA: Poly vinyl alcohol
siRNA: Small interfering RNA
qRT-PCR: Quantitative real time PCR
ePAT: Extension poly(A) test
HCD: Higher-energy collisional dissociation
FDR: False discovery rate
GO: Gene Ontology
RNA-seq: RNA sequencing
SEM: Standard error of the mean
2PN: Two pronuclei
tPNf: Time to pronuclei fading
t2, t3, t4, t5, and t8: Time in hours post-tPNf for the embryo to reach the 2-, 3-, 4-, 5-, and 8-cell stages
tM: Time for compaction
tB: Time for the blastocoel to reach greater than or equal to half the volume of the embryo
AMPK: AMP-activated protein kinase
DEPs: Differentially expressed proteins
MOS: Moloney sarcoma oncogene
KD: Knockdown
OE: Overexpression
ZAR1: Zygote arrest-1
4E-T: EIF4E-transporter protein
YBX1: Y-box binding protein-1
4E-BPs: EIF4E-binding proteins
