# Supplementary figures and images for: Cordycepin delays postovulatory aging of oocytes through inhibition of maternal mRNAs degradation via DCP1A polyadenylation suppression
Source: Cell Mol Life Sci. 2023 Nov 25;80(12):372. doi: 10.1007/s00018-023-05030-0 (PMC10674002; doi:10.1007/s00018-023-05030-0)

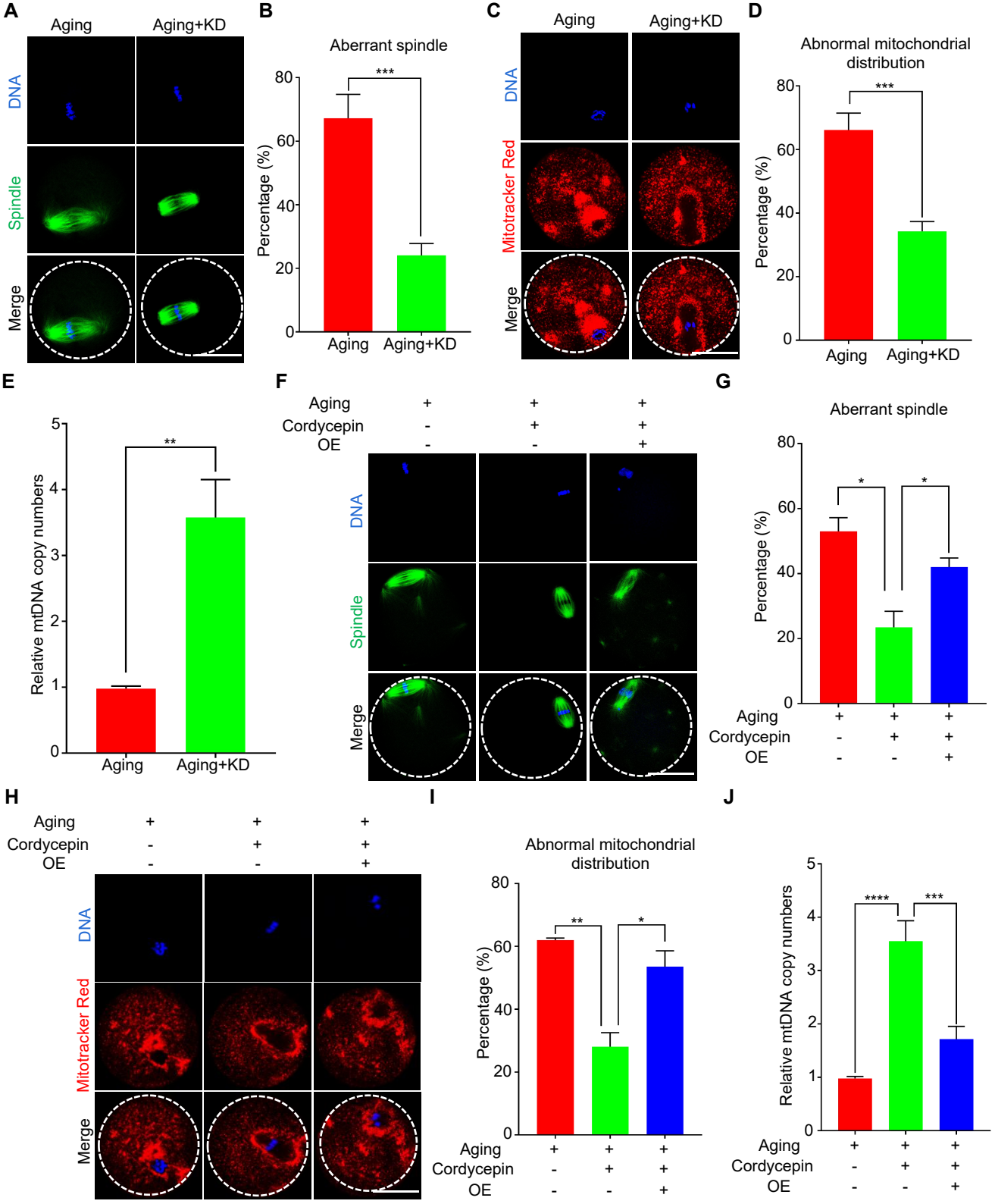

Supplement: Supplementary file 1 — Supplementary file1 Figure S1. Effect of cordycepin supplementation on the parthenogenetic embryonic developmental potential of postovulatory aging oocytes (A) Representative images of 2-cell embryos and blastocysts from fresh, aging and cordycepin-treated groups. Scale bar, 100 μm. (B) The rates of 2-cell embryos and blastocysts were recorded in fresh (n = 135), aging (n = 170) and cordycepin-treated (n = 176) groups. A total of 28 mice were used. Data was presented as mean percentage (mean ± SEM) from four independent experiments. Statistical analysis were performed with one-way ANOVA with Tukey's post hoc test. *P < 0.05, **P < 0.01 (PDF 1133 KB) [file 18_2023_5030_MOESM1_ESM.pdf]

**A**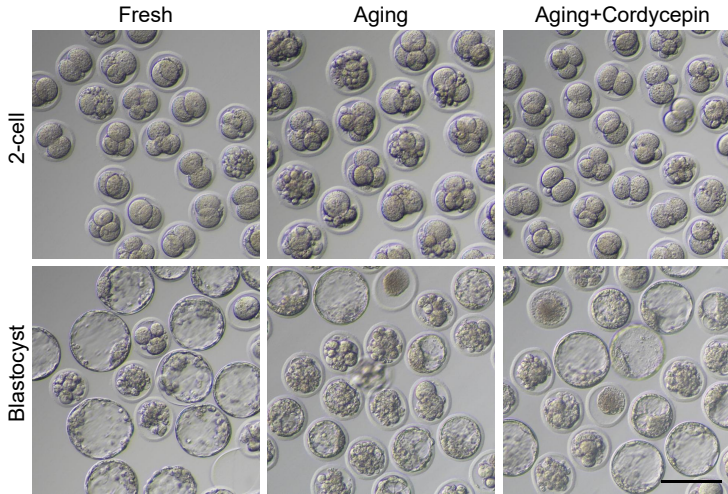**B**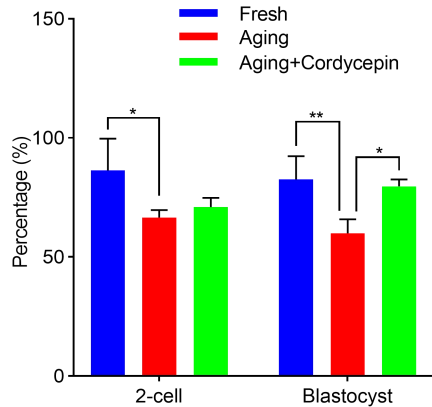

Supplement: Supplementary file 5 — Supplementary file5 Figure S2. Gene expression patterns to the corresponding 11 cordycepin-inhibited decay proteins (A) Transcript levels of the corresponding 11 cordycepin-inhibited decay proteins examined by RNA-seq in fresh, aging, and cordycepin-treated oocytes. (B) Quantitative RT-PCR results showing the expression level of the corresponding 11 cordycepin-inhibited decay proteins in fresh, aging, and cordycepin-treated oocytes. A total of 21 mice were used. Data was presented as mean percentage (mean ± SEM) from three independent experiments. Statistical analysis were performed with one-way ANOVA with Tukey's post hoc test. *P < 0.05, **P < 0.01, ***P < 0.001, ****P < 0.0001 (PDF 460 KB) [file 18_2023_5030_MOESM5_ESM.pdf]

**A**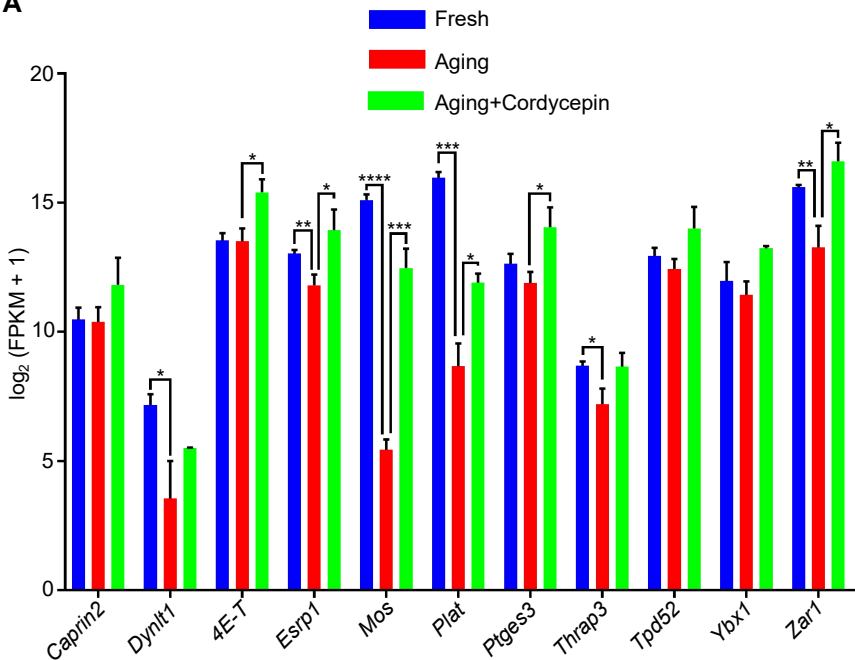**B**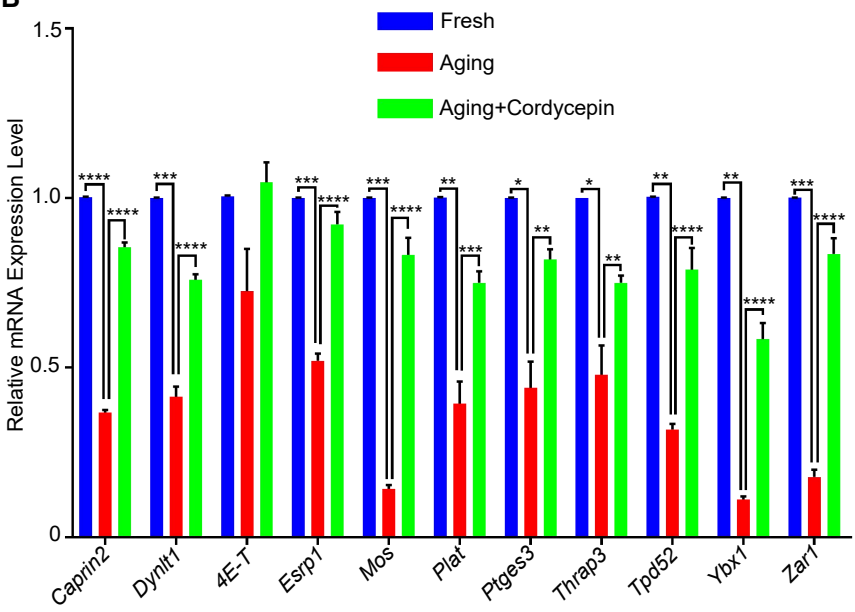

Supplement: Supplementary file 6 — Supplementary file6 Figure S3. Cordycepin inhibits the degradation of MOS protein in aging oocytes (A) Representative images of MOS expression in fresh, aging, and cordycepin-treated oocytes. Scale bar, 50 μm. (B) Fluorescence intensity of MOS signals was measured in fresh (n = 15), aging (n = 14) and cordycepin-treated (n = 1¬¬7) oocytes. (C) Protein levels of MOS examined by Western blot analysis in fresh, aging, and cordycepin-treated oocytes. 200 oocytes for each group were collected and immunoblotted for MOS and β-ACTIN. (D) Quantitative analysis of MOS protein levels from western blot. Data of (B) and (D) were presented as mean percentage (mean ± SEM) from three independent experiments. Number of mice used in (B) and (C) were 6, and 75, respectively. Statistical analysis were performed with one-way ANOVA with Tukey's post hoc test. *P < 0.05, **P < 0.01, ****P < 0.0001. (PDF 468 KB) [file 18_2023_5030_MOESM6_ESM.pdf]

**A**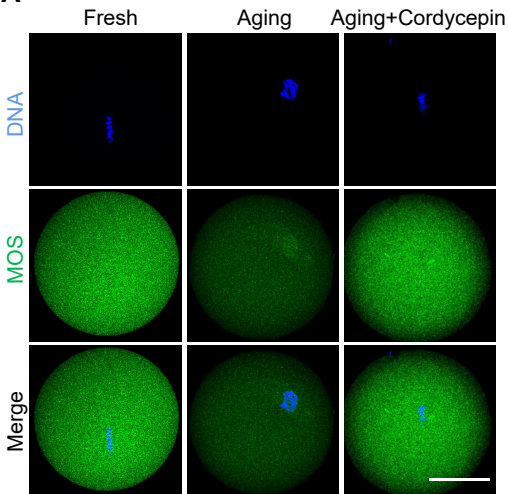**B**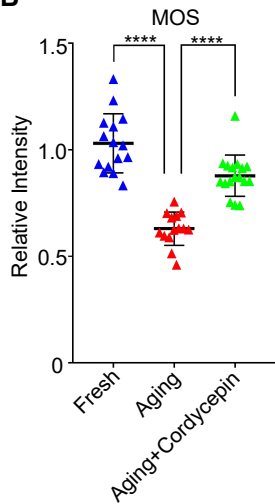**C**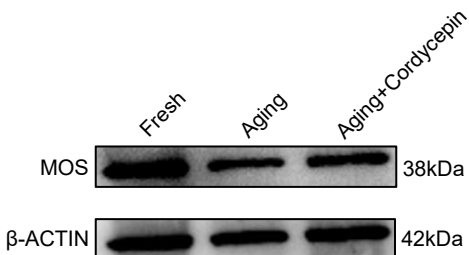**D**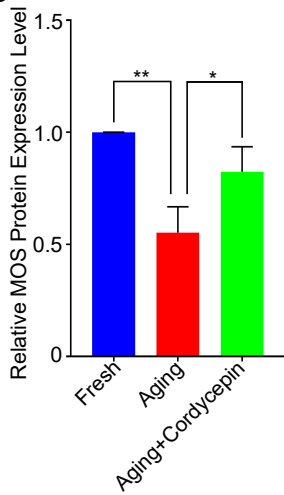

Supplement: Supplementary file 7 — Supplementary file7 Figure S4. Cordycepin improves the MAPK3/1 pathway in aging oocytes. (A) Representative images of p-MAPK3/1 expression in fresh, aging, and cordycepin-treated oocytes. Scale bar, 50 μm. (B) Fluorescence intensity of p-MAPK3/1 signals was measured in fresh (n = 13), aging (n = 14) and cordycepin-treated (n = 17) oocytes. (C) Protein levels of p-MAPK3/1 examined by Western blot analysis in fresh, aging, and cordycepin-treated oocytes. 200 oocytes for each group were collected and immunoblotted for p-MAPK3/1 and MAPK3/1. (D) Quantitative analysis of p-MAPK3/1 protein levels by Western blot. (E) Representative images of p-MAPK3/1 expression in aging, cordycepin-treated, U0126 (1 μM), U0126 (1 μM) + cordycepin, U0126 (3 μM) and U0126 (3 μM) + cordycepin oocytes. Scale bar, 50 μm. (F) p-MAPK3/1 fluorescence intensity in aging (n = 17), cordycepin (n = 22), U0126 (1 μM) (n = 18), U0126 (1 μM) + cordycepin (n = 17), U0126 (3 μM) (n = 14) and U0126 (3 μM) + cordycepin (n = 15) oocytes. (G) The rate of fragmentation was recorded in aging (n = 97), cordycepin (n = 92), U0126 (1 μM) (n = 73), U0126 (1 μM) + cordycepin (n = 117), U0126 (3 μM) (n = 75) and U0126 (3 μM) + cordycepin (n = 83) oocytes. (H) The rate of blastocysts was recorded in aging (n = 120), cordycepin (n = 91), U0126 (1 μM) (n = 113), U0126 (1 μM) + cordycepin (n = 120), U0126 (3 μM) (n = 106) and U0126 (3 μM) + cordycepin (n = 114) oocytes. Data of (B), (D), (F), (G) and (H) were presented as mean percentage (mean ± SEM) from three independent experiments. Number of mice used in (B), (D), (F), (G) and (H) were 6, 75, 9, 24, 24 and 27, respectively. Statistical analysis were performed with one-way ANOVA with Tukey's post hoc test. *P < 0.05, **P < 0.01, ***P < 0.001, ****P < 0.0001, ns: no significance (PDF 1065 KB) [file 18_2023_5030_MOESM7_ESM.pdf]

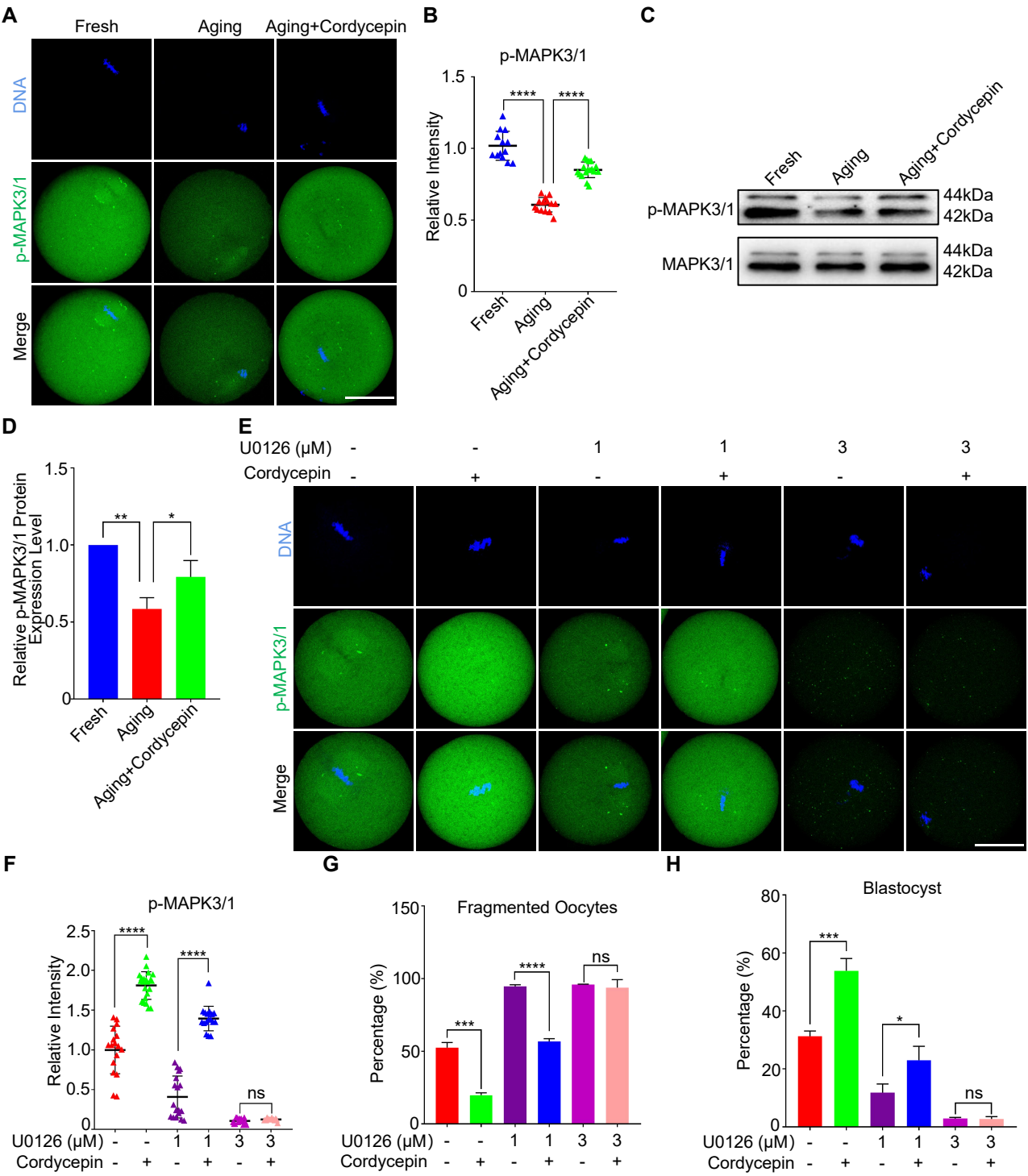

Supplement: Supplementary file 8 — Supplementary file8 Figure S5. Dcp1a knockdown and overexpression in oocytes. (A) Quantitative RT-PCR results showing the expression level of the Dcp1a gene in aging and DCP1A-KD aging oocytes. (B) Fluorescence intensity of DCP1A signals was measured in aging and DCP1A-KD aging oocytes. Scale bar, 50 μm. (C) Fluorescence intensity of DCP1A signals was measured in aging (n = 28) and DCP1A-KD aging (n = 21) oocytes. (D) Quantitative RT-PCR results showing the expression level of the Dcp1a gene in aging and DCP1A-OE aging oocytes. (E) Fluorescence intensity of DCP1A signals was measured in aging and DCP1A-OE aging oocytes. Scale bar, 50 μm. (F) Fluorescence intensity of DCP1A signals was measured in aging (n = 19) and DCP1A-OE aging (n = 19) oocytes. Data of (A), (C), (D) and (F) were presented as mean percentage (mean ± SEM) from three independent experiments. Number of mice used in (A), (C), (D) and (F) were 15, 6, 15 and 6, respectively. Statistical analysis were performed with Student's t test (two-tailed). ****P < 0.0001 (PDF 1777 KB) [file 18_2023_5030_MOESM8_ESM.pdf]

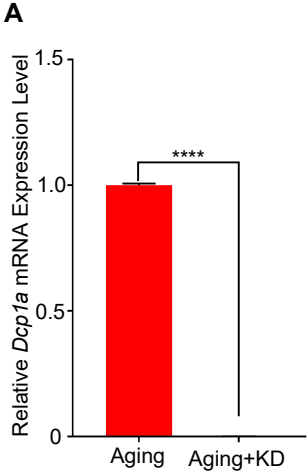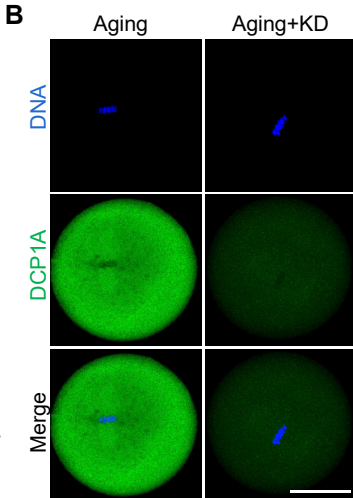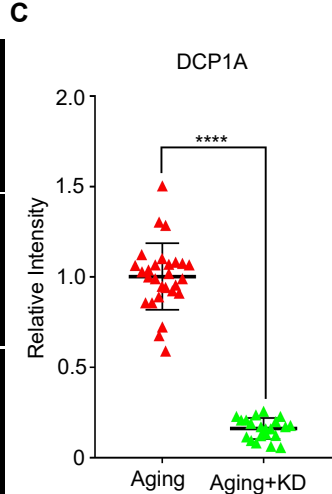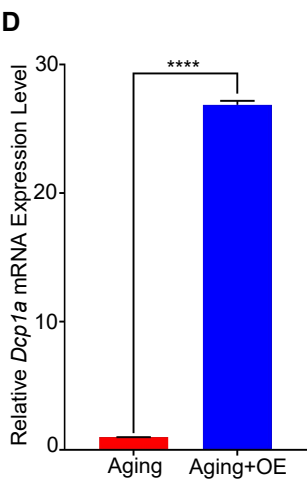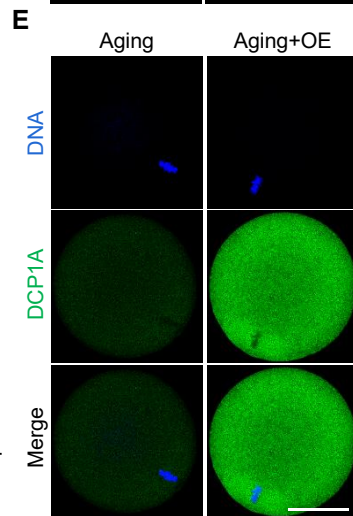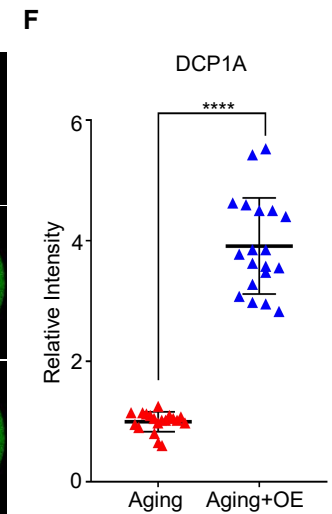

Supplement: Supplementary file 9 — Supplementary file9 Figure S6. Effect of DCP1A-KD and DCP1A-OE on the spindle assembly and mitochondrial distribution in aging oocytes. A Representative images of spindle morphologies in aging and DCP1A-KD aging oocytes. Oocytes were immunostained with α-tubulin-FITC antibody to visualize the spindles and counterstained with Hoechst to visualize the chromosomes. Scale bar, 50 μm. (B) The rate of aberrant spindle was recorded in aging (n = 45) and DCP1A-KD aging (n = 58) oocytes. (C) Representative images of the mitochondrial distribution in aging, and DCP1A-KD aging oocytes. Scale bar, 50 μm. (D) The rates of abnormal distribution of mitochondria in aging (n = 49) and DCP1A-KD aging (n = 49) oocytes. (E) mtDNA copy numbers of aging (n = 15), and DCP1A-KD aging (n = 15) oocytes. (F) Representative images of spindle morphologies in aging, cordycepin and cordycepin + DCP1A-OE oocytes. Oocytes were immunostained with α-tubulin-FITC antibody to visualize the spindles and counterstained with Hoechst to visualize the chromosomes. Scale bar, 50 μm. (G) The rate of aberrant spindle was recorded in aging (n = 42), cordycepin (n = 40) and cordycepin + Dcp1a-OE (n = 38) oocytes. (H) Representative images of the mitochondrial distribution in aging, cordycepin and cordycepin + DCP1A-OE oocytes. Scale bar, 50 μm. (I) The rates of abnormal distribution of mitochondria in aging (n = 44), cordycepin (n = 47) and cordycepin + DCP1A-OE (n = 48) oocytes. (J) mtDNA copy numbers of aging (n = 15), cordycepin (n = 15) and cordycepin + DCP1A-OE (n = 15) oocytes. Data of (B), (D), (E), (G), (I) and (J) were presented as mean percentage (mean ± SEM) from three independent experiments. Number of mice used in (B) & (D), (C), (G) & (I) and (J) were 9, 6, 15 and 6, respectively. In (B), (D) and (E), statistical analysis were performed with Student's t test (two-tailed); and in (G), (I) and (J) one-way ANOVA with Tukey's post hoc test was used for statistical analysis. *P < 0.05, **P < 0.01, ***P [file 18_2023_5030_MOESM9_ESM.pdf]
